# Incidentally Detected Pulmonary Carcinoid Tumorlet With Coexisting Carcinoid Tumorlet in the Mediastinal Lymph Node in a Patient With Lung Cancer

**DOI:** 10.1155/crpu/9910270

**Published:** 2025-08-21

**Authors:** Bhavesh Mohan Lal, Michella K. Whisman, Matthew A. Steliga, Konstantinos Arnaoutakis

**Affiliations:** ^1^Department of Internal Medicine, University of Arkansas for Medical Sciences, Little Rock, Arkansas, USA; ^2^Department of Pathology, University of Arkansas for Medical Sciences, Little Rock, Arkansas, USA; ^3^Department of Surgery, University of Arkansas for Medical Sciences, Little Rock, Arkansas, USA

## Abstract

Pulmonary carcinoid tumorlets are a cluster of neuroendocrine cells in the lung, which invade the basement membrane and are less than 5 mm in size. While similar to carcinoid tumors in all regards, they are differentiated from carcinoid tumors purely based on their size. They are generally considered benign, but lymph nodal involvement has been described in the past. It is unclear if lymph nodal involvement is due to metastasis to the lymph node or synchronous de novo proliferation of neuroendocrine cells in the lymph node as well. Here, we describe a patient with adenocarcinoma of the lung who was incidentally detected to have a pulmonary carcinoid tumorlet and a carcinoid tumorlet in the mediastinal lymph node.

## 1. Introduction

Pulmonary neuroendocrine cells are specialized epithelial cells that are dispersed throughout both lungs, mainly located in the bronchial walls [1]. Proliferation of neuroendocrine cells can lead to a spectrum of disorders, including diffuse idiopathic pulmonary neuroendocrine cell hyperplasia (DIPNECH), carcinoid tumorlets, or carcinoid tumors. Carcinoid tumorlets and carcinoid tumors are characterized by invasion of the basement membrane, while the basement membrane is preserved in patients with DIPNECH [1, 2].

The differentiation between carcinoid tumorlets and carcinoid tumors is purely based on their size. Carcinoid tumorlets are less than 5 mm in size, and carcinoid tumors are more than 5 mm in size. Otherwise, both these conditions share strong histological, ultrastructural, and immunohistochemical similarities [3].

Pulmonary carcinoid tumorlets occur most often in female patients in their 50s or 60s, usually on the right side, often incidentally detected on autopsy or on histopathological examination of a surgical specimen resected for other reasons [3–5]. Since carcinoid tumorlets are often asymptomatic and their diagnosis requires a biopsy, they are often missed. The true incidence and prevalence of this condition are unknown [1].

Pulmonary carcinoid tumorlet is a benign condition; however, rare lymph nodal involvement has been described in case reports [6–11]. It is not clear if this lymph nodal involvement represents a true metastasis or a synchronous de novo proliferation of neuroendocrine cells in the lymph node. Here, we describe a patient with adenocarcinoma of the lung who was also incidentally detected to have a coexisting carcinoid tumorlet in the lung and in the mediastinal lymph node.

## 2. Case Description

A male patient in his 60s, with a 50-pack-year smoking history, presented to our emergency room with seizure-like activity and a fall. He was incidentally detected to have a mass in the upper lobe of his right lung on the CT angiography of the head and neck performed in the emergency room. Contrast-enhanced CT of the chest was performed to better visualize the lesion, which showed a well-defined, multilobulated mass lesion arising from the right upper lobe, measuring up to 3.3 cm, with no mediastinal adenopathy. A CT-guided biopsy was performed, and the histopathology established a diagnosis of adenocarcinoma of the lung. Whole body PET-CT and MRI of the brain did not show any metastatic disease or FDG avid lymph nodes. He was diagnosed with Stage IB (T2aN0M0) adenocarcinoma of the lung.

**Figure 1:**
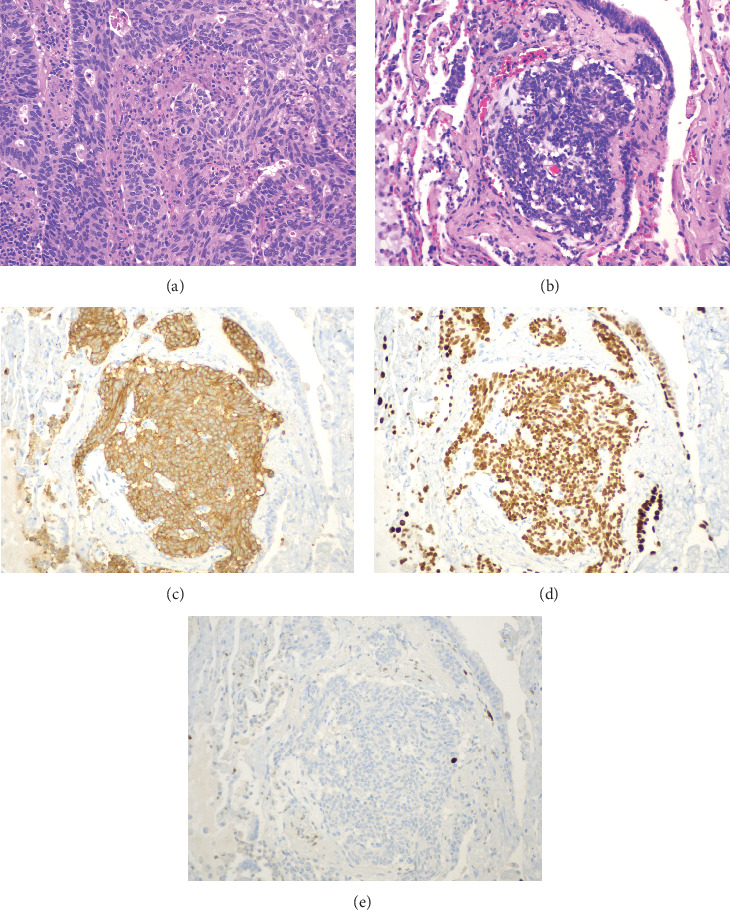
Histopathology and immunohistochemistry of adenocarcinoma of the lung with pulmonary carcinoid tumorlet. (a) Morphology of the invasive adenocarcinoma (H&E, 200x). (b) Pulmonary carcinoid tumorlet (H&E, 200x). (c) Pulmonary carcinoid tumorlet (synaptophysin, 200x). (d) Pulmonary carcinoid tumorlet (TTF-1, 200x). (e) Pulmonary carcinoid tumorlet (Ki-67, 200x).

**Figure 2:**
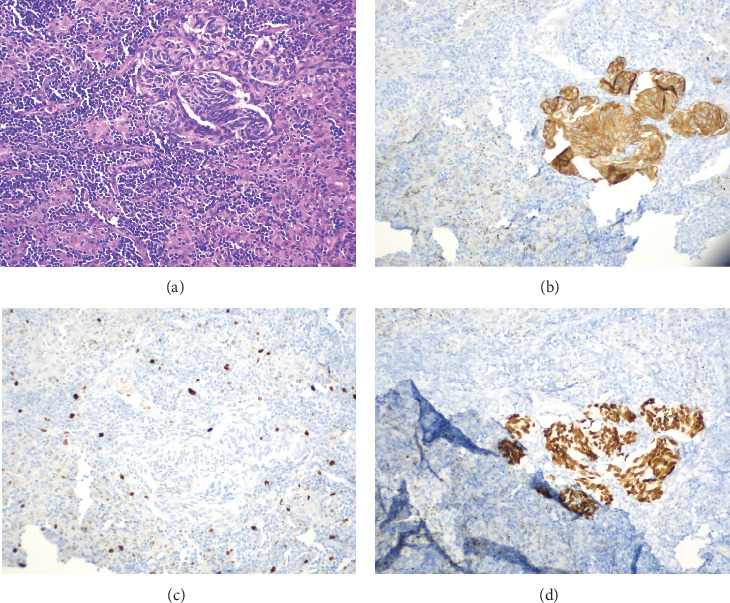
Carcinoid tumorlet in the mediastinal lymph node. (a) Lymph node carcinoid tumorlet (H&E, 200x). (b) Lymph node carcinoid tumorlet (synaptophysin, 200x). (c) Lymph node carcinoid tumorlet (TTF-1, 200x). (d) Lymph node carcinoid tumorlet (Ki-67, 200x).

Video-assisted thoracoscopic surgery with right upper lobectomy and right mediastinal lymphadenectomy was performed. Specimen from right upper lobectomy showed poorly differentiated adenocarcinoma (TTF-1 positive, synaptophysin negative, chromogranin negative, and high Ki-67 proliferation index) with negative surgical margins and negative peribronchial lymph nodes. Additionally, separate clusters of bland cells with stippled chromatin measuring up to 0.7 mm in size were seen. By immunohistochemistry, these cells were diffusely positive for synaptophysin, CD56, and TTF-1, negative for PAX-8 and CDX-2, and had a low (< 1%) proliferation index on Ki-67; these findings were consistent with a carcinoid tumorlet (see Figure 1). Lymph nodes from 4R, 7, 9R, 10R, and 11R did not show any evidence of metastatic disease. However, one of the three lymph nodes from level 7 showed a focus of carcinoid tumorlet measuring 0.3 mm in size, which was again positive for synaptophysin, CD56, and TTF-1 and negative for PAX-8 and CDX-2 on immunohistochemistry with Ki-67 proliferation index < 1% (see Figure 2).

Given high-risk features and poor histological grade, the patient was given the option of adjuvant chemotherapy, which he declined. The patient continues to follow with us for routine posttherapy surveillance with no recurrence of disease after 5 months of surgery.

## 3. Discussion

Carcinoid tumorlets have traditionally been diagnosed postmortem in patients with chronic pulmonary inflammation and fibrosis. If diagnosed antemortem, a carcinoid tumorlet is most often an incidental finding on histopathology after surgical resection of the lung for adenocarcinoma or for bronchiectasis with recurrent infections, although it has rarely been described with squamous cell carcinoma as well [2, 4, 5, 12–14]. Jin et al. reported a case series of four patients with DIPNECH and multiple carcinoid tumorlets. Three out of the four patients had chronic inflammation and fibrosis with bronchiectasis, and one patient had adenocarcinoma of the lung with no bronchiectasis [15].

In our case report, we describe a patient with adenocarcinoma of the lung, who was incidentally detected to have a coexisting carcinoid tumorlet in the lung and in the mediastinal lymph node on histopathology of the surgical specimen. The pulmonary tumorlet was < 1 mm in size with a low proliferation index, raising a question of whether it was truly responsible for the metastasis to the lymph node. The possible explanations for the lymph node involvement include a metastatic deposit in the lymph node from the carcinoid tumorlet in the lung, synchronous de novo carcinoid tumorlet in the lung and in the mediastinal lymph node, or a metastatic deposit in the lymph node due to another coexisting carcinoid tumor.

In our patient, no other nodules were seen on multiple CT and PET/CT imaging of the chest, making a metastatic deposit in the lymph node due to coexisting another carcinoid tumor unlikely.

Generally, carcinoid tumorlets are considered benign. Aubry et al. looked at 28 patients with multiple carcinoid tumors or tumorlets in surgical specimens and did not find metastases in any patient with carcinoid tumorlets in the absence of carcinoid tumors [11]. But a metastatic deposit in the lymph node from a carcinoid tumorlet has previously been described (see Table 1). D'Agati and Perzin incidentally detected multiple carcinoid tumorlets in the lung with metastasis to a peribronchial lymph node in a patient with bronchiectasis [7]. Similarly, Cureton and Hill, Demirağ and Altınok, and Dogan et al. found lymph nodal metastasis due to multiple pulmonary carcinoid tumorlets in the setting of bronchiectasis and recurrent infections [8–10]. Immune system dysregulation in the context of chronic inflammation and/or cancer, allowing early metastasis, cannot be excluded.

Li et al. described four patients with carcinoid tumorlet in intrathoracic lymph nodes found during surgical resection of adenocarcinoma of the lung. Interestingly, carcinoid tumorlet in the lung was seen in only one out of the four (25%) patients. All these patients had localized lung cancer with no lymph nodal involvement or distant metastasis. One of the four patients developed a 2-cm typical carcinoid of the right pleura, on the same side as intrathoracic lymph node involvement, 5 months after the initial surgery [6]. While it would be interesting to see if all patients with intrathoracic lymph nodal carcinoid tumorlet develop carcinoid tumors over time, most of them have coexisting primary lung malignancy, making longer follow-up difficult due to poor survival rates.

In a patient undergoing surgical resection of primary adenocarcinoma of the lung, Al-Ayoubi et al. incidentally detected DIPNECH with multiple carcinoid tumorlets and a carcinoid tumorlet on the parietal pleura. In the absence of a tumor > 5 mm and the absence of lymph node metastases, the authors assumed it likely to be a de novo growth in the parietal pleura rather than a metastatic deposit [12]. It is interesting to note that while DIPNECH and carcinoid tumorlets are much more common in females (females represent ~90% of cases), the reported cases of extrapulmonary carcinoid tumorlets have been predominantly in males (seven males out of 10 patients—70%) [1].

De novo synchronous carcinoid tumorlet in the lymph node is a possibility. An observation supporting this hypothesis is that N1 lymph nodal involvement in typical carcinoids has no impact on prognosis [16]. The de novo growth can either happen from proliferation of de novo neuroendocrine cells or transdifferentiation of other cells or neuroendocrine differentiation of uncommitted stem cells. Hermann et al. searched for neuroendocrine cells in gastrinoma triangle lymph nodes at autopsy in 20 cases that had no abdominal tumors using gastrin antibody and synaptophysin antibody. They found neuroendocrine cells in five out of 20 (25%) cases, indicating that neuroendocrine cells may sometimes be normally seen in gastrinoma triangle lymph nodes [17]. Neuroendocrine cells have not been demonstrated in the intrathoracic lymph nodes yet. While Li et al. found individual neuroendocrine cells in the intrathoracic lymph nodes, these patients also had coexisting carcinoid tumorlets in the lymph nodes, and it would be difficult to conclude that neuroendocrine cells are normally seen in intrathoracic lymph nodes based on this observation alone [6].

It is still not clear if carcinoid tumorlets in lymph nodes represent a true metastasis or a de novo proliferation of neuroendocrine cells in the intrathoracic lymph nodes. It is also unclear if the presence of a carcinoid tumorlet in an intrathoracic lymph node portends a higher chance of developing a carcinoid tumor in the future. It is important to find answers to these questions for better management of patients and to further expand our knowledge about carcinoid tumorlets.

## Figures and Tables

**Table 1:** Case reports and case series of patients with extrapulmonary carcinoid tumorlets in the absence of carcinoid tumors in the lung.

**Author**	**Year**	**Age (years)/sex**	**Primary pulmonary disease**	**Pulmonary neuroendocrine pathology**	**Extrapulmonary carcinoid tumorlets**
Al-Ayoubi et al. [12]	2014	81/female	Adenocarcinoma of the lung	DIPNECH with multiple carcinoid tumorlets	Parietal pleura without lymph node involvement
Cureton and Hill [8]	1955	36/male	Bronchiectasis	Multiple carcinoid tumorlets	Multiple bronchopulmonary and one trachea–bronchial lymph node
D'agati and Perzin [7]	1985	38/male	Bronchiectasis	Multiple carcinoid tumorlets	One peribronchial lymph node
Demirağ and Altınok [9]	2003	38/male	Bronchiectasis	Multiple carcinoid tumorlets	One peribronchial lymph node
Dogan et al. [10]	2009	34/male	Bronchiectasis	Multiple carcinoid tumorlets	One peribronchial lymph node
Li et al. [6]	2010	59–81/2 males, 2 females	Adenocarcinoma of the lung in all 4 patients	Only 1 out of 4 patients had carcinoid tumorlet in lung	Lymph node involvement in all 4 patients
Our case	2024	60s/male	Adenocarcinoma of the lung	Single carcinoid tumorlet	Single mediastinal lymph node involvement

## Data Availability

The data that support the findings of this study are available from the corresponding author upon reasonable request.
